# Shedding Light on the Pharmacological Interactions between μ-Opioid Analgesics and Angiotensin Receptor Modulators: A New Option for Treating Chronic Pain

**DOI:** 10.3390/molecules26206168

**Published:** 2021-10-13

**Authors:** Kornél Király, Dávid Á. Karádi, Ferenc Zádor, Amir Mohammadzadeh, Anna Rita Galambos, Mihály Balogh, Pál Riba, Tamás Tábi, Zoltán S. Zádori, Éva Szökő, Susanna Fürst, Mahmoud Al-Khrasani

**Affiliations:** 1 Department of Pharmacology and Pharmacotherapy, Faculty of Medicine, Semmelweis University, Nagyvárad tér 4, P.O. Box 370, H-1445 Budapest, Hungary; karadi.david_arpad@med.semmelweis-univ.hu (D.Á.K.); zador.ferenc@pharma.semmelweis-univ.hu (F.Z.); mohammadzadeh.amir@med.semmelweis-univ.hu (A.M.); galambos.anna@pharma.semmelweis-univ.hu (A.R.G.); balogh.mihaly@med.semmelweis-univ.hu (M.B.); riba.pal@med.semmelweis-univ.hu (P.R.); zadori.zoltan@med.semmelweis-univ.hu (Z.S.Z.); furst.zsuzsanna@med.semmelweis-univ.hu (S.F.); 2 Department of Pharmacodynamics, Faculty of Pharmacy, Semmelweis University, Nagyvárad tér 4, H-1089 Budapest, Hungary; tabi.tamas@pharma.semmelweis-univ.hu (T.T.); szoko.eva@pharma.semmelweis-univ.hu (É.S.)

**Keywords:** µ-opioid analgesics, angiotensin receptors, chronic pain, neuropathic pain

## Abstract

The current protocols for neuropathic pain management include µ-opioid receptor (MOR) analgesics alongside other drugs; however, there is debate on the effectiveness of opioids. Nevertheless, dose escalation is required to maintain their analgesia, which, in turn, contributes to a further increase in opioid side effects. Finding novel approaches to effectively control chronic pain, particularly neuropathic pain, is a great challenge clinically. Literature data related to pain transmission reveal that angiotensin and its receptors (the AT1R, AT2R, and MAS receptors) could affect the nociception both in the periphery and CNS. The MOR and angiotensin receptors or drugs interacting with these receptors have been independently investigated in relation to analgesia. However, the interaction between the MOR and angiotensin receptors has not been excessively studied in chronic pain, particularly neuropathy. This review aims to shed light on existing literature information in relation to the analgesic action of AT1R and AT2R or MASR ligands in neuropathic pain conditions. Finally, based on literature data, we can hypothesize that combining MOR agonists with AT1R or AT2R antagonists might improve analgesia.

## 1. Introduction

Among different types of chronic pain, neuropathic pain is defined by the International Association for the Study of Pain (IASP) as pain caused by a lesion or disease of the somatosensory nervous system (IASP 2012). There are many available treatment approaches for the management of neuropathic pain. Yet, despite these advances, it remains an unmet medical need because most of the treatment approaches intended to halt this pain condition are not effective enough or sometimes effective but limited by side effects. Thus, finding new targets and innovative future strategies that might help to improve neuropathic pain control are of clinical need.

µ-Opioid receptor (MOR) agonists are the mainstay treatment for different forms of chronic pain [1,2,3,4]. However, their efficacy in the management of neuropathic pain is a long-standing question of debate. Yet, international guidelines restrict opioids to second- or third-line therapy, with no clear consensus on their effect [5,6,7]. MOR agonists with significantly higher intrinsic efficacy than morphine produced acceptable analgesia in preclinical models of neuropathic pain [8,9]; however, this has not been successfully utilized clinically because clinical trials showed controversial results related to their efficacy and liability for side effects [10,11,12,13]. In response to this argument, many studies have been conducted to increase the efficacy and decrease the side effects of opioids when used in the management of neuropathic pain. Some of the encouraging strategies that aim to improve the analgesic effect and decrease the side effects of currently used analgesics, such as opioids, are based on combining two or more different agents. However, so far, clinical research data that is based on combination strategies have not met expectations [14]. Chaparro et al., reviewed clinical trials on the efficacy and safety of various agent combinations for neuropathic pain [14]. Their analysis revealed that the combination of opioids with gabapentin was significantly better than gabapentin alone in reducing the symptoms. However, the number of treated patients that was required for a single patient to benefit was still 9.5, and significantly more participants experienced side effects and thus dropped out of the studies with opioids plus gabapentin than with gabapentin alone [14]. On the other hand, studies assessing the effects of opioids in combination with other sensory-sensitization blocking agents could be of high clinical value. Thus, continuing preclinical research based on the application of multi-target drugs or combination strategies that involve implementing different agents might bring a new treatment option for neuropathic pain. In the former case, for instance, applying opioid receptor ligands that display agonist and non-opioid effects, such as tapentadol, display both the MOR agonist and norepinephrine reuptake inhibitory effects in the same molecule [15]. Recently, our group reported on the promising effect of the combination of glycine transporter 1 and 2 inhibitors in the management of neuropathic pain evoked by sciatic nerve ligation [16]. In such a strategy, we need to consider how the individual drugs affect pain transmission.

Accumulating evidence has proven that drugs affecting the renin–angiotensin system can modulate pain transmission [17,18,19,20,21,22,23,24,25,26,27,28,29,30,31,32,33,34]. Recent studies have also shown that drugs mimic or antagonize angiotensin type 1 and 2 (AT1R and AT2R) receptor-mediated actions do produce a beneficial analgesic effect in rodent models of chronic pain types [17,20,22,28,29,35,36,37,38]. The analgesic effect of ligands affecting angiotensin receptors in neuropathic pain is explained by the contribution of these receptors to neuroregeneration and neuroprotection—partially by reducing neural inflammatory processes [18,24,37,39,40,41]. Nevertheless, much remains unclear regarding the role and clinical utility of these receptors in analgesia. 

This review briefly highlights how the effect of MOR agonist-induced analgesia is altered under neuropathic pain conditions, showing the advantages and drawbacks, as well as principal factors that negatively impact the analgesic effect of MOR analgesics in this pain entity. The next sections review the implication of angiotensin and its receptors in chronic pain, particularly that associated with neuropathy, and also the neuroanatomical overlap between MORs and angiotensin receptors in relation to pain. Finally, according to the reviewed data, perspectives on the future drug combination-based research strategy to treat neuropathic pain are provided. With respect to angiotensin IV and its receptor, the presence of the peptide has been reported in human dorsal root ganglia (DRG) and trigeminal nucleus (TG) [42,43]. However, there are little data related to their analgesic effect. Thus, they will not be discussed in the present review.

## 2. The Opioid System and the µ-Opioid Receptor in Different Pain Entities

**Figure 1:**
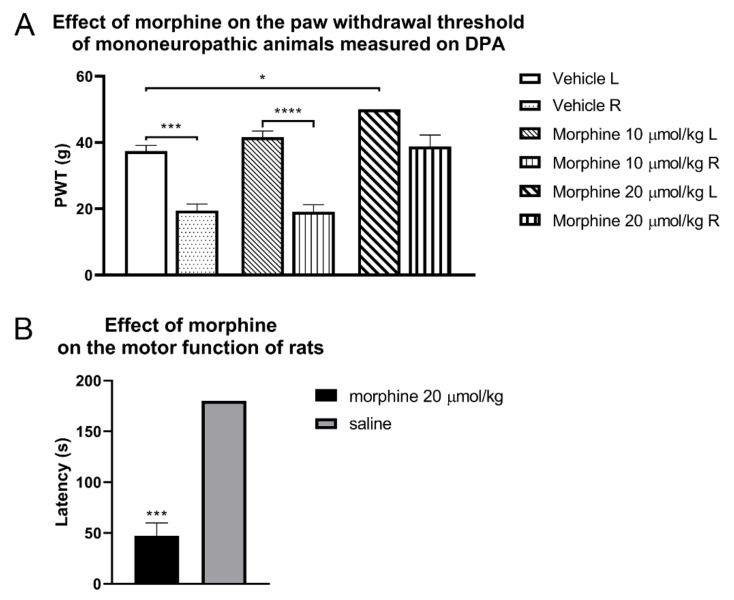
(**A**) The analgesic effect of morphine measured on a dynamic plantar aesthesiometer (DPA) test at 30 min, after s.c. administration to mononeuropathic animals. Columns represent the paw withdrawal threshold of the animals in grams ± S.E.M. Asterisks indicate the significant differences between treatment groups or operated (R) and non-operated (L) hind paws (* *p* < 0.05; *** *p* < 0.001 and **** *p* < 0.0001). Statistical differences were determined with one-way ANOVA and Tukey post-hoc test. Data represent means ± S.E.M (n = 5–12 per group). (Karádi, D.Á.; Al-Khrasani, M.; unpublished data). (**B**) Effect of the systemic administration of morphine to the motor function of rats. Columns represent the time latency of the animals in sec ± S.E.M. at 30 min post-treatment in the rotarod test. Asterisks indicate the significant differences compared to the saline group (one-way ANOVA, Newman–Keuls post-hoc test; *** *p* < 0.001). In each treatment group, 4–7 animals were used. These results were adopted from our previous work [16].

The opioid system is a physiological system for controlling pain, but it also participates in addictive behaviors and immune defense, among others. Mammalian endogenous opioid peptides and exogenous natural, semisynthetic and synthetic opioid agonists can produce their effects through the activation of opioid receptors, namely µ-(MOR), δ-(DOR), and κ-(KOR) opioid receptors. Opioid receptors belong to the class A G-proteins of the pertussis toxin-sensitive Gi/Go family. Their effectors include adenylyl cyclase, N- and L-type Ca^2+^ channels, and inwardly rectifying K^+^ channels. Upon activation, adenylyl cyclase and Ca^2+^ channels are inhibited, whereas K^+^ channels are activated. Thus, both the limitation of Ca^2+^ entry and the hyperpolarization of the cells may give a tenable explanation for the inhibition of transmitter release at pain traffic points [44,45]. With respect to pain, central MORs are the principal target for mediating the analgesic effects of opioids. As in MOR-knockout mice, selective MOR agonists failed to produce analgesia as well as MOR-induced opioid side effects, such as respiratory depression, gastrointestinal transit inhibition, and addiction liability [46,47]. Since the identification of functional peripheral MORs, it has become obvious that the analgesic effects of opioids do not solely depend on MORs at the central nervous system (CNS) [48]. It is worth noting that achieving peripheral analgesia requires prerequisite factors that are related both to the physicochemical properties of opioid analgesics (limited CNS penetration) and pain entity. In the case of the latter, the pathological state of pain largely reflects the effects of opioid analgesics. In inflammatory or acute non-inflammatory pain, MORs number is increased or maintained at normal level, respectively [9,48,49,50]. Several opioid researchers have proven that functional MORs in the periphery are targetable, particularly in inflammatory pain types [51,52,53,54]. However, under neuropathic pain conditions, several studies have demonstrated the downregulation of MORs in the dorsal spinal cord and DRG [9,55]. The efficacy of currently available MOR agonists in neuropathic pain is a question of debate. Taken together, in cases of acute or inflammatory pain types, opioid analgesics can provide adequate pain control, which is somewhat hampered by above mentioned unwanted effects. However, in the case of neuropathic pain, the desired analgesia itself is often unachievable, consequently demanding dose-escalation, therefore causing more pronounced side effects (Figure 1A) (Karádi and Al-Khrasani, unpublished data) and (Figure 1B) (adopted from our previous work [16]).

For restoring the effect of opioids in neuropathic pain, many attempts have been focused on the mechanisms related to changes in the number of functional MORs on sensory neurons in subjects with painful neuropathy. In our and other studies carried out in rats with neuropathic pain induced either by streptozotocin (STZ) or chronic constriction injury (CCI), the number of MORs was found to be decreased in DRG and spinal tissue [9,56,57]. This reduction in MOR number was accompanied by a decrease in the analgesic effects of opioids.

## 3. Angiotensin Receptor Mimetics and Antagonists in Relation to Pain

### 3.1. Endogenous Angiotensin Ligands and Angiotensin Receptors 

Components of the renin–angiotensin system (RAS) have been previously reviewed or discussed extensively [19,24,58,59,60,61,62,63,64]. Nevertheless, the main findings are briefly summarized here for an overview. Among the endogenous peptides of the RAS, neuronal angiotensin II (Ang II) is the most significant in relation to pain. Ang II is an octapeptide derived from the inactive precursor angiotensinogen, which is initially cleaved by renin, resulting in the inactive intermediate angiotensin I (Ang I). Ang II is cleaved from Ang I by the angiotensin-converting enzyme 1 (ACE1). Ang II equally binds to and activates the AT1R and AT2R (see later on). Another relevant endogenous peptide of the RAS to this review is angiotensin 1-7 (Ang (1-7)), which is cleaved by the angiotensin-converting enzyme 2 (ACE2) from Ang II or by ACE1 from Ang I via the intermediate angiotensin 1-9. Ang (1-7) activates the Ang (1-7) receptor or MAS receptor, but it can also bind with lower affinity to AT2R. 

There are four angiotensin receptor types known so far within the RAS; namely angiotensin II type 1 and 2 receptors, the angiotensin IV receptor, and the Ang (1-7) receptor or MAS receptor (abbreviated as AT1R, AT2R, AT4R, and AT7R or MASR, respectively). Additionally, in mice and rats, two AT1R isoforms have been identified, namely AT1aR and AT1bR [65,66]. In relation to the RAS, this review will focus on data of AT1R, AT2R, and MASR, with respect to pain, particularly from preclinical studies. They all belong to the rhodopsin-like G-protein coupled receptor family (GPCR); however, they differ significantly in terms of activation of signaling pathways and cellular and tissue distribution patterns. The latter will be discussed in detail in a separate section. The AT1R is a prime example of a GPCR that upon activation can be dependent and independent from heterotrimeric G-proteins, allowing the receptor to have a wide range of signaling responses to Ang II. In terms of G-protein dependent signaling pathways, the AT1R couples to multiple types of Gα, (Gq/11, Gi, G12, and G13), but it also includes the activation of small G-proteins. G-protein independent signaling of AT1R involves β-arrestin 1 and 2, tyrosine kinase-related signaling, reactive oxygen species signaling, receptor-interacting scaffold proteins, or heterodimerization with AT2R or MASR. In the case of AT2R, signaling pathways are still not fully elucidated, in spite of the intensive research. In fact, it is one of the least understood areas of the renin–angiotensin system. Most interestingly, it fails to demonstrate classic GPCR signaling features, such as affecting second messengers (e.g., cAMP, diacylglycerol) or the lack of phosphorylation-induced receptor desensitization, or internalization in most tissue types. However, it has been proven that AT2R is sensitive to GTPγS and pertussis toxin in rat locus coeruleus, indicating Gi/o coupling [67]. AT2R can also stimulate protein phosphatases and nitric oxide production. In addition, AT2R mediates the inactivation of mitogen-activated protein kinase (MAPK) inhibition which is important in the induction of apoptosis [60,67]. The AT2R and Ang II interaction leads to neurite formation and growth via the modulation of polymerized β-tubulin, microtubule-associated proteins (MAP), the activation of the p42/p44 MAPK phosphorylation of trkA. MASR, similar to AT1R and AT2R, can couple to many downstream signaling pathways via Ang (1-7) activation. These include the activation of phospholipase C and A2, arachidonic acid release, or calcium-independent nitric oxide synthase activation. MASR also modulates several kinase-related pathways/effectors, such as the p38 MAPK, ERK1/2, phosphatidylinositol 3-kinase/Akt, RhoA, and cAMP/PKA, in different cell lines. MASR was also demonstrated to constitutively couple to Gαi, Gαq, and Gα12/13 [63]. On the other hand, similar to AT2R, in most cases, MASR fails to induce the conventional G-protein mediated signaling response, defined by the levels of classical second messengers, such Ca^2+^, or inositol trisphosphate (IP3), despite belonging to the GPCR family.

### 3.2. AT1 and AT2 Receptor Agonists

Following the discovery of the neuronal RAS, numerous studies have reported on the implication of AT1R/AT2R agonists on nociception [27,30,33,68,69,70,71,72,73,74,75,76]. In spite of the high number of studies conducted, literature data remain highly controversial. Some publications describe the analgesic activity of AngII, AngIII, or renin on acute pain tests following central (intracerebroventricular [27,69,71,72,76] or intrathecal [33]) administration. These reports proposed different possible mechanisms of action behind the observed effects. Many of them indicate the role of the endogenous opioid system as the analgesic activity of test compounds was naloxone-sensitive [27,33,69,71,72]. Next, Shimamura et al., suggested a kinetic interaction between AngIII and met-enkephalin, namely the inhibition of cleavage of the latter [71]. Georgieva et al., found that AngII administered intracerebroventricularly (icv.) produced an antinociceptive effect in the acetic-acid writhing pain model, yet the AngII-induced antinociception was blocked by PD123319, an AT2R selective antagonist but not by losartan, an AT1R antagonist [75]. In this study, the authors concluded that AT2Rs but not AT1Rs are involved in the mechanism behind the analgesic action in acute inflammatory pain. Since then, studies assessing the effects of RAS peptides (angiotensinogen, AngI, AngII, or AngIII) microinjected into different regions of the periaqueductal gray (PAG) were conducted in rats. In these studies, all test peptides were proven to be analgesic on the tail-flick assay, and their effect was AT1R or AT2R antagonist reversible [77]. Another observation is that spontaneously hypertensive rats show longer latency on the hot plate but not on the tail-flick test, when compared to wild-type animals. Moreover, this increase in latency can be reversed by orally administered captopril or losartan, but not by antihypertensive agents which are acting on targets other than the RAS [73]. In contrast to the above-mentioned studies, Cridland et al., reported that AngII failed to show either anti- or pronociceptive effect [72]. However, at present, we cannot judge this issue because, to the best of our knowledge, there is no other study that supports Cridland’s observations. It is also worth considering the article of Pavel et al., which examined the effect of AngII and losartan in rats undergoing CCI. In these animals, intraperitoneal AngII was found to be pronociceptive in the von Frey test (mechanical stimuli), constant hot- and cold-plate tests and decremental cold plate test (thermal stimuli). Losartan fully reversed the effect of AngII in case of mechanical stimuli, partially reversed it in case of constant cold-plate test, but further aggravated it in the decremental cold plate test. In the incremental hot plate test, the pain threshold was unchanged both following AngII or AngII + losartan administration [78]. The differences observed in this study between the effect of angiotensin in response to constant or decremental/incremental thermal stimuli is difficult to explain.

Further on, the direct pronociceptive activity of AngII and AngIII was described as spontaneous painful behavior (scratching) was observed following intrathecal administration [40,41]. It is worth noting that the study of Cridland et al., showed neither anti- nor pronociceptive action of AngII, whereas Nemoto and coworkers reported a pronociceptive action. Despite the similar administration route, the phenotype of the animals, as well as the dose applied, was different in these studies [40,41,73]. Therefore, further studies are needed to elucidate the effect of AngII at the spinal level. Indirectly supporting the pronociceptive action of AngII, Kaneko et al., reported icv. administered AngII to attenuate the analgesic activity of morphine in a dose-dependent manner in hot plate and tail pinch tests [69]. Similarly, Yamada et al., found that icv. administrated AngII or the AT2R agonist novokin decreased the antinociceptive effect of morphine in the tail-pinch test [79]. Shepherd et al., also reported an increased mechanical but not thermal allodynia following intraplantar AngII administration in mice after spared nerve injury (SNI) [80]. 

There is large literature data on neural regeneration and differentiation mediated by the AT2R, which were recently reviewed by Danigo et al. [24]. From this aspect, activating the AT2R induces positive changes in terms of neural injury. This neuroprotective action linked to the AT2R has been associated with an increase in neuronal BDNF expression by several reports. The AT2R agonist “compound 21” (C21) has been reported to increase neurite growth following spinal nerve injury [81] and to improve survival while attenuating post-stroke neurological deficit in mice [82]. Under these conditions, the common feature was an increase in neuronal BDNF expression. In contrast, increasing BDNF level is not necessarily beneficial in cases of peripheral nerve injury from the aspect of pathological pain, since Madara et al., showed that BDNF could induce glutamate release by enhancing the action of presynaptic NMDA receptors [83]. BDNF release governs the spinal long-term potentiation of C-fibers [84]. Long-term potentiation and a consequently increased glutamatergic tone, involving the increased activity of spinal NMDA receptors, are hallmarks of neuropathic pain or other chronic pain states [85,86]. Furthermore, Chen et al., proved that spinal NMDA receptor-potentiation on primary afferents in neuropathic pain could be blocked either by the BDNF scavenger trkB-Fc or by the trkB receptor antagonist ANA-12 [87]. The contribution of BDNF to pain was validated by Sikandar et al., where they demonstrated that the conditional knockout of BDNF from mouse sensory neurons results in unchanged response to most acute pain types and displayed hypoalgesia in chronic inflammatory or neuropathic pain [88]. 

### 3.3. MAS Receptor Agonists

Primarily the Ang (1-7)-MASR branch of RAS acts as an antagonist of the AngII-AT1R activity. The activity linked to AT2Rs is similar in general; however, with respect to pain transmission, this is not the case. The possible analgesic effect of Ang (1-7) was investigated following mostly local (intraplantar [21,23] or intrathecal [34,89,90,91,92,93]) administration. Studies using intraplantar administration reported that Ang (1-7) attenuated PGE2 [21,23,90,91] or carrageenan [23] induced inflammatory mechanical hyperalgesia. The antihyperalgesic effect of Ang (1-7) was lost in MASR KO mice [23] and was reversible by MASR, nNOS, guanylyl cyclase, or ATP-sensitive potassium channel blockers [94] as well as by different adrenergic antagonists [21], but not by naloxone [95].

Intrathecal administration of Ang (1-7) resulted in a decrease in spontaneous nociceptive behavior induced by intrathecal AngII [91], AngIII [92], substance P or NMDA [34]. Furthermore, intrathecal Ang (1-7) showed an antiallodynic and antihyperalgesic effect in neuropathic pain induced by CCI [89], STZ [90], or genetic model of diabetes (ob/ob mice) [93]. Moreover, several authors reported that Ang (1-7) effectively decreased the pathological increased p38 phosphorylation in the spinal cord [90,91,92,96]. Similar results were reported following intrathecal administration of ACE2 activator DIZE, namely reduced nociceptive behavior in the formalin test and decreased spinal p38 phosphorylation [96]. On the other hand, intraplantar Ang (1-7) was ineffective in the treatment of CCI induced neuropathic pain [23].

The effect of systemic (ip.) administration of Ang (1-7) on bone cancer pain was investigated by Forte et al., In this model, Ang (1-7) reduced spontaneous pain reactions, increased von Frey threshold and tail immersion latency following acute or chronic administration. The authors reported no anti-tumor activity [97].

### 3.4. AT1 and AT2 Receptor Antagonists

**Figure 2:**
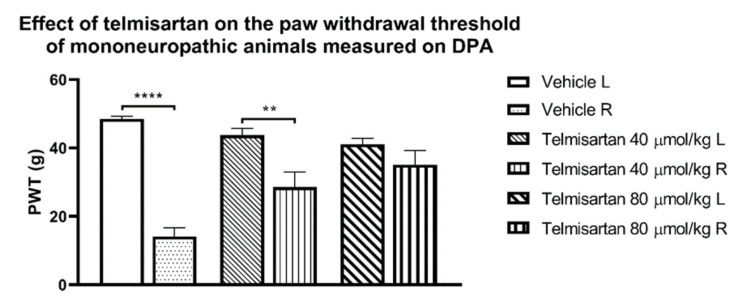
The analgesic effect of telmisartan measured on a dynamic plantar aesthesiometer (DPA) test at 120 min, after p.o. administration to mononeuropathic animals induced by partial sciatic nerve ligation rat model described by Seltzer et al. [98]. Columns represent the paw withdrawal threshold (PWT) of the animals in grams ± S.E.M. Asterisk indicates the significant differences between treatment groups or operated (R) and non-operated (L) hind paws (** *p* < 0.01 and **** *p* < 0.0001). Statistical differences were determined with one-way ANOVA and Tukey post-hoc test. Data represent means ± S.E.M (n = 5 per group). (Karádi, D.Á.; Al-Khrasani, M.; unpublished data).

A growing body of literature data supports that antagonists of the AT1R, such as losartan, candesartan, or telmisartan, among others, display analgesic action in different pain models, including acute thermal, inflammatory, or neuropathic pain [17,23,30,35,36,39,40,41]. With respect to the analgesic effect of telmisartan, our unpublished results also support such findings because it could reduce the partial sciatic nerve CCI-induced allodynia after systemic administration in rats (Figure 2) (Karádi and Al-Khrasani, unpublished data)).

In addition, intrathecal administration of losartan has been reported to block AngII-induced spontaneous pain [39], both phases of formalin test [41], and STZ-induced allodynia [99]. On the other hand, microinjection of AT1R and AT2R antagonists into the PAG has been reported to aggravate incisional allodynia [26,77]. Local administration of losartan was also investigated by Costa et al., In this study, intraplantar (ipl.) losartan effectively reversed prostaglandin E2 (PGE2) and carrageenan-induced mechanical hyperalgesia but was ineffective in CCI induced neuropathic pain [23]. In contrast, numerous publications have reported that systemic administration of AT1R antagonists to be beneficial [17,20,35,36,79]. Most of these reports suggest that blocking AT1R could also attenuate the inflammatory reaction in DRG [35,36] or the sciatic nerve [17] and elevate the decreased BDNF level in the sciatic nerve [17] following neuronal damage. 

Bessaguet et al., investigated the effect of candesartan on resiniferatoxin-induced neurotoxic thermal hypoalgesia in mice and proved that intraperitoneal candesartan was able to reverse the evoked hypoalgesia in this assay, yet the same effect was achieved following the treatment with AT2R antagonist, EMA200 (PD123319). The authors proposed that candesartan may increase the AT2R binding of endogenous AngII, thus lowering the thermal threshold of animals. This proposal is further supported by the lack of efficacy of candesartan in AT2R KO mice [20]. In agreement with these results, Hashikawa-Hobara et al., reported that hypoesthesia caused by fructose induced diabetes was reversible by orally administered candesartan [100]. Obagata et al., showed that intrathecal losartan can attenuate the allodynia evoked by STZ in mice. In addition, they found that Ang II, as well as ACE expression, were increased, indicating the involvement of AngII in neuropathic pain conditions. It has also been reported that candesartan is capable of inducing neuroprotective, anti-inflammatory, and pro-angiogenetic effects accompanied by an increase in BDNF expression [101,102]. In these studies, the beneficial effects of AT1R antagonism were reversible by the AT2 receptor antagonist, EMA200 [101,102]. Similar to the above-mentioned studies, the authors hypothesized that AT1R antagonism causes a shift in endogenous AngII binding from the AT1R to the AT2R, thus indirectly causing AT2R activation.

There are numerous studies indicating that AT2R antagonism can be beneficial in treating different pain entities. In case of inflammatory pain types, the proposal that reduction in hyperinnervation can attenuate pain is in agreement with literature data [103,104]. Chakrabarty et al., reported that EMA200 reduced thermal hyperalgesia, mechanical allodynia, and pathological hyperinnervation of inflamed tissue in a model of inflammatory pain induced by complete Freund’s Adjuvant (CFA) [18,22]. The same compound was also effective in the treatment of cancer-induced bone pain, which is mostly an inflammatory pain type, strongly depending on local inflammatory mediators [105]. The most clinically promising results, however, came from the investigation of the analgesic effect of EMA200 and its analogs in neuropathic pain, partially contradicting the above-mentioned data [28,29,37,38,80,106,107,108]. These include rodent models of mononeuropathic pain and even human clinical trials. AT2R antagonists were shown to be able to attenuate mechanical [37,38,81,107,108] and cold [107] allodynia in different mononeuropathic models, such as CCI or SNI. Moreover, the effect of EMA200 was validated on complex behavioral pain assays as well [109]. The most clinically relevant result, however, is that the analgesic effect of EMA401, the orally available analog of EMA200, was tested in clinical trials for postherpetic neuralgia [28,29] and diabetic neuropathy [28]. The efficacy in attenuating symptoms of the patients enrolled was acceptable in both conditions; however, two of the three studies were prematurely terminated because of preclinical data on the possible hepatotoxic effect of the test compound upon long-term administration [28]. There is no clear consensus whether AT2Rs are expressed on sensory neurons creating a direct pharmacological target for analgesia [18,37,38,106,107,110], or the observed beneficial effect is mediated by immune cells infiltrating injured nerves [80,107]. The neuro-immune cross-talk proposed by the latter studies was recently reviewed by Balogh et al. [19].

## 4. Neuroanatomical Distribution of the µ-Opioid and Angiotensin Receptors in Areas Related to Pain

### 4.1. The µ-Opioid Receptor

The neuroanatomical distribution of the MOR is now well-established by immunohistochemistry, autoradiography, in situ hybridization, and fluorescence techniques [109,110,111,112,113]. Accordingly, MORs can be found at supraspinal, spinal, and peripheral levels [114,115,116]. MORs are enriched in the descending pain modulatory pathway, involving the periaqueductal gray (PAG) matter, rostral ventromedial medulla (RVM), locus coeruleus (LC), and the dorsal horn of the spinal cord [115,117]. In addition, they can be found in brain regions that are strongly related to pain perception and integration, such as the cerebral cortex, thalamus, striatum, amygdala, hippocampus, nucleus accumbens, and the ventral tegmental area (VTA) [115,117]. Within the dorsal horn of the spinal cord, MORs are densely localized in the lamina I-II superficial layers on interneurons and projection neurons [115,118]. The dorsal root ganglia are also a significant locus for MORs attributed to pain [115,119]. MORs can also be found on C- and A-fibers and near primary afferent nociceptors [117].

### 4.2. Angiotensin Receptors and Endogenous Angiotensin Ligands 

The components of neuronal angiotensin system are found in anatomical regions hosting different key points in pain pathways, including the dorsal horn of the spinal cord, dorsal root ganglia (DRG and identical structures, such as the spinal trigeminal tract and trigeminal ganglion), or peripheral nerves. Angiotensinogen mRNA can be found ubiquitously in the mammalian brain [120], spinal cord [99], and almost all cells in the DRG [42,43]. The angiotensinogen level in the CNS is not affected by STZ treatment-induced diabetes; however, it is elevated following peripheral inflammation [22,121].

There are contradictory data in the literature about the localization of neuronal renin, the primary activating enzyme of the renin–angiotensin system [42,43,100]. AngI mRNA is present in the human DRG and trigeminal ganglion (TG) [42,43], whereas its protein form was described in rat DRG [121]. AngII was found in rat and human DRG [18,37,43,106,107], TG [42], neurons, satellite cells, and CD3+ T-cells [106]. The colocalization of AngII alongside components involved in pain sensation, such as substance P (SP) and vanilloid transient receptor potential channels, was reported as well on small and medium neurons [18,37,42,43,106]. In rodent, AngII can be found ubiquitously in the spinal cord; its level was highest in the superficial laminae of the dorsal horn, which could suggest a possible role of AngII in nociception [41,99]. Furthermore, AngII levels have been reported to be increased following mono- or polyneuropathic pain evoked by CCI [106] or STZ, respectively [41,99]. Furthermore, this change in AngII levels was also seen in pain conditions induced by intraplantar formalin injection [41,99] or in bone cancer pain [105]. 

With respect to the receptors, several studies have reported on the distribution of AT1R on key points related to nociceptive transmission both in mice [39,40] and rats [31,36,43,122,123,124,125,126,127,128,129]. These areas include sciatic nerve [31,127,130], DRG [36,43,123,125,127,128,129,130,131], and spinal cord [22,39,40,129,132]. Moreover, it can be found in different brain regions, such as the spinal trigeminal tract and raphe nuclei [122]. These data also provide strong evidence on a large amount of AT1aR, and smaller amounts of AT1bR mRNA [43,127,129,132], and the receptor protein [31,36,39,40,123,125,127,128,129] was also shown in the mentioned regions. In the DRG, the receptor protein was found on satellite cells and neurons of all sizes with a greater extent on smaller ones [36,110,128,130]. In the spinal cord, similarly to AngII, AT1R level was the highest in the superficial dorsal horn [39,128]. 

In contrast to AT1R, AT2R localization and the above-mentioned function in relation to nociception are controversial subjects. At present, little data are available on the ganglional or sensory neural expression of AT2R as many of the currently commercially available AT2R antibodies used for immunohistochemistry seem to show inappropriate specificity [131]. Therefore, it is important to evaluate the results of studies using antibodies with appropriate criticism—especially in case of earlier works.

Early autoradiographic studies found significant inhibition of AngII binding by AT1R but not by AT2R antagonists on the sciatic nerve, spinal cord, and (upper cervical) sensory ganglion [31,128]. AT2 mRNA was found in the DRG and sciatic nerve of rats [43,127]. The receptor protein was found by many research groups on neurons (IB4+ [132]), satellite cells [106,127], and CD3+ T-cells [106] and in the rat DRG as well [101,106,107,110,125,130]. Indeed, in a few studies, the AT2 antibody specificity was verified on AT2R KO mice, further reinforcing the results [37,123]. On the other hand, Shepherd and colleagues were not able to find AT2R mRNA or protein in the DRG of mice or humans [80]. In their study using Agtr2^GFP^ reporter mice, the AT2 positivity in the sciatic nerve was detectable and increased after SNI but because of macrophage infiltration instead of neural expression. Taken together, Shepherd’s group claims that AT2R is not expressed on sensory neurons involved in nociception [107]. In contrast, Benitez et al., found AT2 immunoreactivity in rat DRG mostly on non-peptidergic (IB4+) C- and Aδ-fibers showing high colocalization to AT1 yet using an antibody with specificity verified on AT2R KO mice. In their study, the level of AT2 increased in an inflammatory state following treatment with CFA [123]. It is important to mention that mice were used in the study conducted by Shepherd in contrast to rats used by Benitez. A very recent review published in 2021 by Danigo et al., provides detail on how to solve this contradiction and lists species differences as well as the possible gene duplication of AT2R (similar to AT1R) in mice which could cause a lack of signal in the reporter mice [24].

Angiotensin-converting enzyme 2 (ACE2) is a carboxypeptidase enzyme regulating the local levels of AngII and Ang 1-7 (metabolizes AngII to Ang 1-7). Its mRNA and protein were found in human DRG samples, colocalizing with nociceptor neuronal markers [133]. It is also expressed in mouse spinal cord, where it is localized on neurons and microglia but not on astrocytes [93]. Finally, MASR expression was shown in rat DRG [91,92], PAG [134] and in mouse spinal cord [93]. However, to the best of our knowledge, the localization of the Ang (1-7) peptide has not been fully described. The neuroanatomical localization of key elements of the RAS and µ-opioid receptors have been summarized in Table 1.

## 5. Possible Link between MOR Analgesics and Ligands Affecting Angiotensin Receptors in Relation to Pain

Rather than dose escalation of MORs analgesics which is associated with an increase in the incidence of side effects, augmenting MORs-mediated analgesia would be an important strategy in the management of neuropathic pain. In regard to the interaction between opioid and angiotensin systems, to the best of our knowledge, the first study published in 1983 by Haulica et al., described that AngII produced naloxone reversible analgesia following icv. administration in rat tail-flick test; therefore, these results showed the implication of endogenous opioid system in the effect of AngII [68]. In a later study, the same research group also reported that naloxone or saralasin attenuates stress analgesia in rats [70]. Based on another study by Han et al., icv. administered AngII was able to reverse the antinociceptive action of sc. morphine [76]. Similarly, Yamada et al., showed that AT2R activation decreases the analgesic effect of morphine [79]. On the other hand, a previous study by Mojaverian et al., reported that orally administered ACE inhibitor enalapril failed to influence morphine analgesia [143]. Recently, Taskiran and Avci reported that systemic captopril alone was able to increase tail-flick and hot plate latency, and it also increased the analgesic effect of systemic morphine. Furthermore, the co-treatment with captopril reduced morphine-induced analgesic tolerance development. Captopril also reduced the inflammatory and endoplasmatic stress response in the DRG caused by acute or chronic morphine treatment [32]. It is important to note however, that ACE inhibition could result in a diverse molecular effect, partly independent from RAS—such as the inhibition of the catabolism of endogenous opioids and peptide mediators, among others. Next, connection between Ang (1-7), MASRs and the opioid system is unclear as to the best of our knowledge there are little data available at present. In this respect, Costa et al., reported that endogenous opioids do not play a role in the analgesic action of Ang (1-7) as it was not sensitive to naloxone [95]. This does not necessarily mean that there are no possible interactions between the two systems. Indeed, there are several reports, indicating opioids are capable of changing physiological parameters, most notably changes in the blood pressure [144,145,146,147,148] or drinking-response to AngII [149,150,151]. However, regarding the relationship between RAS and the opioid system only a small proportion of these address the role of interactions in analgesia. We have summarized the outcomes of relevant studies in Table 2.

**Figure 3:**
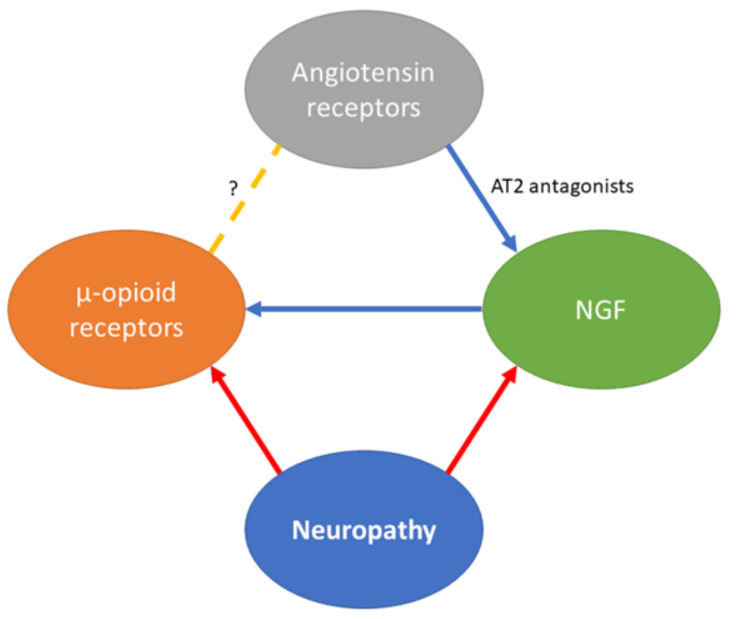
Possible links between neuropathy, the renin–angiotensin system, MORs and NGF. Red arrows indicate a reducing effect, while the blue ones indicate an increasing effect. In neuropathic conditions, the MOR reserve is decreased, resulting in impaired opioid analgesia. The receptor number can be restored by administration of NGF, the level of which is also reduced in the spinal cord in neuropathy. AT2 antagonists are capable of restoring the lowered NGF level, thus possibly restoring the analgesic effect of opioids. To the best of our knowledge, there is no evidence of the direct connection between MORs and the renin–angiotensin system. The figure was constructed based on literature discussed in Section 5.

With respect to neuropathic pain, Khan and coworkers showed that allodynia caused by CCI of the sciatic nerve was attenuated by a systemic single dose of EMA300, a small molecule AT2R antagonist [106]. In this study, the authors also proved that the nerve growth factor (NGF) level was significantly reduced in the ipsilateral lumbar DRGs of neuropathic rats. In addition, treatment with EMA300 could restore the decreased NGF level. Furthermore, several studies have shown that MOR reserve in the spinal cord and DRG is decreased in rodents with neuropathic pain. It is worth noting that administration of exogenous NGF does restore both MOR numbers and their analgesia at main relay points along the pain pathways, such as the spinal cord [58]. These results support a hypothesis on the possible existence of a link between MORs and angiotensin receptor affecting ligands which may provide a new strategy for the treatment of neuropathic pain. Namely, AT2R blockade was reported to restore pathologically decreased NGF levels in neuropathy, which, in turn, could positively influence the MOR number in the DRG and spinal cord, thus restoring the analgesic effect of MOR agonists (Figure 3). An opposing viewpoint is the implication of NGF in pain induction which is not the scope of the present review but has been reported by other researchers [152,153,154]. Finally, whether activation or blockade of AT2R would be of value in managing neuropathic pain, we could propose that AT2R inhibition attenuates pain mediated by largely unidentified pathways. On the other hand, the neural growth and remodeling induced by AT2R activation may be beneficial for neuroregeneration, though undesired effects on the symptoms of neuropathy may occur.

To the best of our knowledge, so far, no publication has investigated the possible connections between the opioid system and the Ang (1-7)—MAS receptor branch of the RAS.

## 6. Concluding Remarks and Future Directions

MOR analgesics alleviate neuropathic pain; however, high doses are needed, which, in turn, result in serious side effects both in preclinical and human studies. Current evidence indicates that AT1, AT2, and MASRs are involved in the control of neuropathic pain; however, their mechanism of action related to neuropathic pain has not yet been fully verified. Nevertheless, AT1, AT2, and MASRs are expressed in key areas related to pain where MORs agonists halt pain sensation. In neuropathic conditions, peripheral and central AT1 blockade and spinal MASRs activation appear to be beneficial. Data on the impact of AT2R in neuropathic pain are contradictory, though its activation or inhibition can result in neuroprotection or analgesia, respectively; however, future studies are needed to justify this issue. So far, there are no angiotensin receptor affecting agents that have been utilized clinically; however, there are clinical studies on AT2R inhibitors that have entered phase II trials but did not proceed further due to their toxicity. It is important to note that these clinical studies prove that such AT2R inhibitors showed equipotent efficacy with gabapentin. In neuropathic pain, the MOR receptors and NGF levels are decreased. Treatment with NGF results in restoring MOR and their analgesic activity in preclinical pain studies. On the other hand, there are studies reporting the increase in pain sensation upon NGF use, which is not the scope of the present review. Furthermore, some studies revealed that angiotensin AT2R inhibitors do increase NGF in neuropathic pain and thus normalize MOR levels. Therefore, we can speculate that drugs affecting angiotensin receptors could restore the effect of MOR analgesics, which results in avoiding dose escalation of opioids upon the treatment of neuropathic pain. Finally, these strategies might offer a bridge upon titration of drugs with delay in onset used in the treatment of neuropathic pain.

## Figures and Tables

**Table 1:** Neuroanatomical distribution of ligands and receptors in the renin–angiotensin system with importance in pain transmission and the µ-opioid receptor (MOR).

Ligand/Receptor	Species	mRNA /Peptide/Protein	Method	Details	Changes		References
					Inflammation	Neuropathy	
**Peripheral nerves**							
**Angiotensinogen**	rat	p	IHC	detected	increased	-	[22]
AT1 receptor	rat	p	autorad	detected	-	-	[31]
	rat	r	PCR	detected	-	increased	[124]
AT2 receptor	rat	p	autorad	not detected	-	-	[31]
	rat	r	PCR	detected	-	increased	[124]
	AgtrGFP reporter mouse	p	reporter mouse	detected on thick non-peptidergic neurons	-	increased (macrophage infiltration)	[107]
MAS receptor	mouse	p	IHC	detected	-	increased	[135]
MOR	rat	p	IHC	detected	increased	-	[136]
	human	p	IHC	detected on CGRP positive skin sensory nerves	no change	-	[137]
**Dorsal root ganglia**							
Angiotensinogen	rat	p	IHC	detected	increased	-	[22]
	rat	r and p	PCR and IHC	detected	-	-	[121]
	rat	r	PCR and ISH	detected on all cells	-	-	[43]
Angiotensin I	human	p	RIA	detected	-	-	[43]
Angiotensin II	rat and human	p	IHC and RIA	colocalized with SP and CGRP	-	-	[43]
	rat	p	IHC	colocalized with neuronal markers	increased (bone metastasis)	-	[105]
	rat	p	IHC and WB	colocalized with SP and NF200	-	increased	[37]
	human	p	IHC	colocalized with TRPV1 on small and medium neurons	-	-	[18]
	rat	p	IHC	on neurons, satellite cells, and T cells	-	increased	[106]
Angiotensin (1-7)	human	p	IHC	not detected	-	-	[18]
AT1 receptor	rat	r	PCR	detected	-	no change	[124]
	rat	r	PCR	detected	-	-	[43]
	rat	p	IHC	detected on Schwann cells, satellite cells, and neurons	-	decreased (DM)	[127]
	rat (isolated neurons)	r and p	PCR, WB, and RB	detected	decreased (TNFα)	-	[129]
	rat	p	IHC	detected on small and large neurons	-	increased	[125]
	rat	p	IHC	detected on neurons and satellite cells	-	-	[36]
	rat	p	IHC	detected on all neurons, higher expression on small	increased on large neurons	-	[123]
AT2 receptor	rat	r	PCR	detected	-	increased	[124]
	rat	r and p	PCR and IHC	detected	-	-	[121]
	rat	r	PCR	detected	-	-	[43]
	rat	p	IHC	detected on Schwann cells, satellite cells, and neurons	-	increased (DM)	[127]
	rat (cell culture)	p	WB	detected	-	increased (DM)	[100]
	rat	p	IHC	colocalized with neural markers	-	-	[37,105]
	rat (neonatal)	r and p	PCR, WB, and IHC	detected on IB4+ neurons	-	-	[132]
	rat	p	IHC	detected on neurons, satellite cells, and T-cells	-	no change	[106]
	rat	p	IHC	detected on all neurons, mostly non-peptidergic C and Aδ, high colocalization with AT1	increased	-	[123]
	AgtrGFP reporter mouse and human	r and p	PCR and reporter mouse	not detected	-	-	[80]
	AgtrGFP reporter mouse	p	reporter mouse	not detected	-	no change	[107]
MAS receptor	rat	p	IHC	detected	-	-	[95]
	rat	r and p	PCR and WB	detected	-	increased	[89]
	rat	r and p	PCR and WB	detected	-	-	[138]
	mouse	p	WB	detected	increased (bone metastasis)	-	[97]
MOR	rat	p	IHC	detected mainly on small neurons	increased	-	[136]
	rat	p	IHC	detected on small and medium neurons, highly colocalized with CGRP and SP	-	-	[139]
	rat	p	IHC	detected	increased	-	[50]
	rat	r	PCR	detected	increased	decreased	[140]
	human	r	PCR	detected on approx. 50% of neurons, mainly capsaicin-responsive small neurons	-	-	[119]
**Spinal cord**							
Angiotensin II	mouse	p	IHC	detected ubiquitously, highest in laminae I and II	increased	increased	[41,99]
AT1 receptor	rat	p	IHC, autorad, and ISH	detected in the superficial DH and on cholinergic neurons in the VH	-	-	[126,128]
	mouse	p	IHC	detected in the superficial DH	-	-	[39,40]
AT2 receptor	rat	p	IHC	detected in laminae I and II and colocalized with IB4 and SP in	-	-	[123]
	AgtrGFP reporter mouse	p	reporter mouse	detected in the deep DH and VH and colocalized with neuronal markers	-	no change	[107]
MAS receptor	mouse	p	WB	detected	-	-	[93]
	mouse	p	IHC	detected and colocalized with NK1 and NMDA receptors	-	-	[34]
MOR	rat/guinea pig	p	autorad	detected in the superficial dorsal horn	-	-	[113]
	rat	p	IHC	detected on laminae I-II	increased	-	[136]
	rat	p	IHC	present	-	-	[139]
	rat	p	IHC	postsynaptic MOR is restricted to lamina II	-	-	[141]
	rat	p	IHC	detected, half of MOR immunoreactivity in the SC is on primary afferents	-	-	[142]
	rat	r	PCR	detected	no change	no change	[140]
	rat	p	IHC	detected	-	decreased (reversible by NGF)	[57]

Abbreviations: p: peptide/protein; r: mRNA; IHC: immunohistochemistry; autorad: autoradiography; PCR: polymerase chain reaction; ISH: in situ hybridization; RIA: radioimmunoassay; WB: Western blot; DM: diabetes mellitus; DH: dorsal horn; VH: ventral horn; SP: substance P; CGRP: calcitonin gene-related peptide; NF200: neurofilament protein 200; TRPV1: transient receptor potential cation channel subfamily V member 1; IB4: isolectin B4; NK1: neurokinin 1; NMDA: N-methyl D-aspartate. A hyphen indicates no assessment by the indicated studies.

**Table 2:** Reported connections between the opioid and renin–angiotensin systems in relation to pain.

RAS Ligand/Receptor	Method	Outcome	Reference
Angiotensin II	rat tail-flick test	AngII mediated analgesia is reversible by naloxone.	Haulica et al., 1983 [68]
	rat tail-flick test	AngII is able to attenuate morphine analgesia.	Han et al., 2000 [76]
Angiotensin-converting enzyme	rat tail-flick test	ACE-inhibition cannot influence morphine analgesia.	Mojaverian et al., 1984 [143]
	rat tail-flick and hot plate test	ACE-inhibition enhances morphine analgesia and decreases the development of opioid analgesic tolerance.	Taskiran et al., 2021 [32]
	ELISA	ACE-inhibition decreases inflammatory cytokine levels in the DRG of morphine tolerant animals.	Taskiran et al., 2021 [32]
AT2 receptor	mouse tail/pinch test	AT2 activation decreases morphine analgesia	Yamada et al., 2009 [79]
	rat tail-flick test	Saralasin (AT2 partial agonist) decreases stress analgesia.	Haulica et al., 1986 [70]

Abbreviations: ELISA: enzyme-linked immunosorbent assay.
